# Supplementary figures and images for: Evaluation of AAV-DJ vector for retinal gene therapy
Source: PeerJ. 2019 Jan 17;7:e6317. doi: 10.7717/peerj.6317 (PMC6339780; doi:10.7717/peerj.6317)

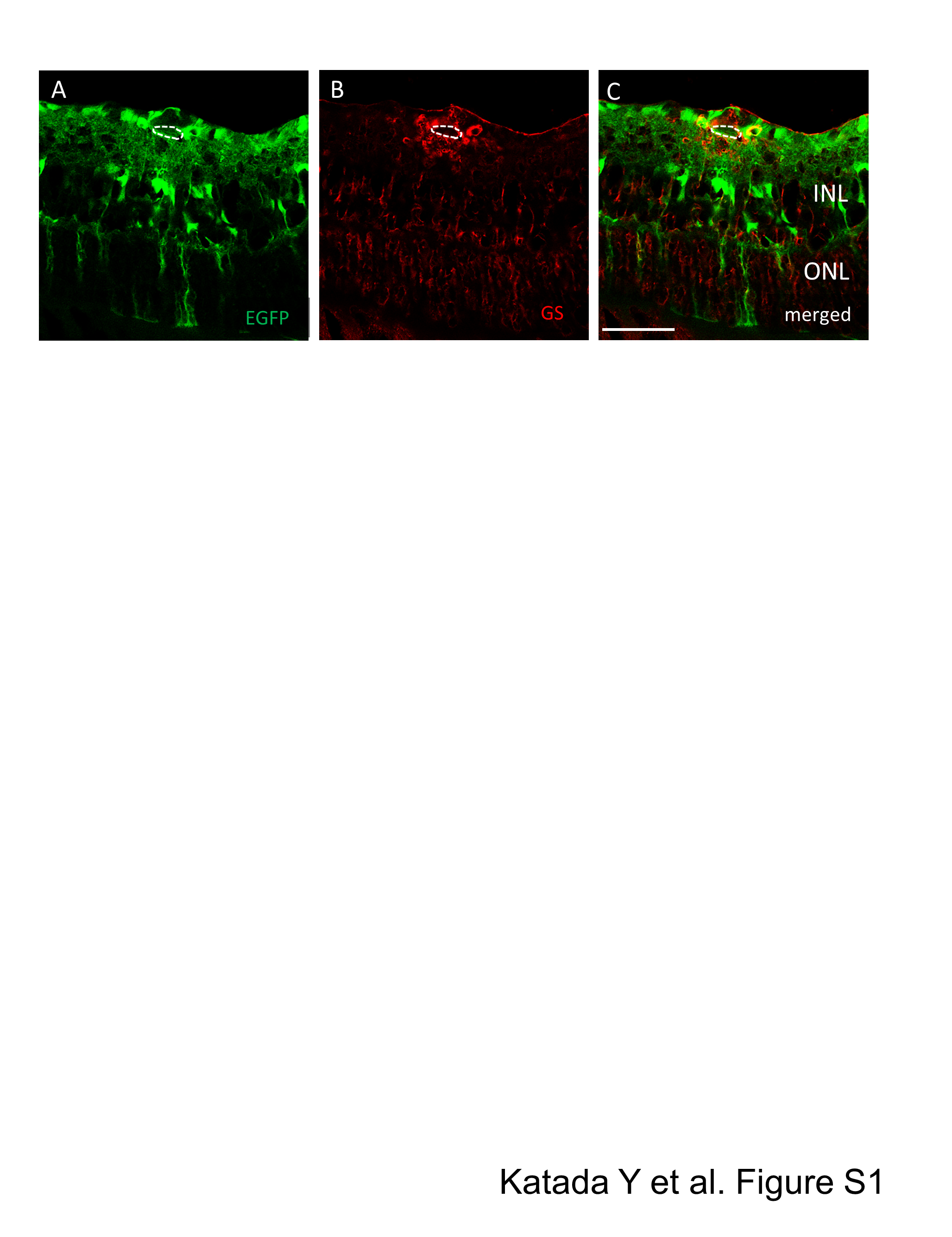

Supplement: Figure S1 — Immunohistochemistry for GS (red) labelling Mueller cells on the transverse retinal cryosection with intravitreal injection of AAV-DJ-CAGGS-EGFP (green). Note that processes of Mueler glia around the blood vessel co-labelled with GS and EGFP. The dotted line indicates a retinal blood vessel. INL, inner nuclear layer; ONL, outer nuclear layer. Scale bars, 50 µm in A–C. [file peerj-07-6317-s001.png]

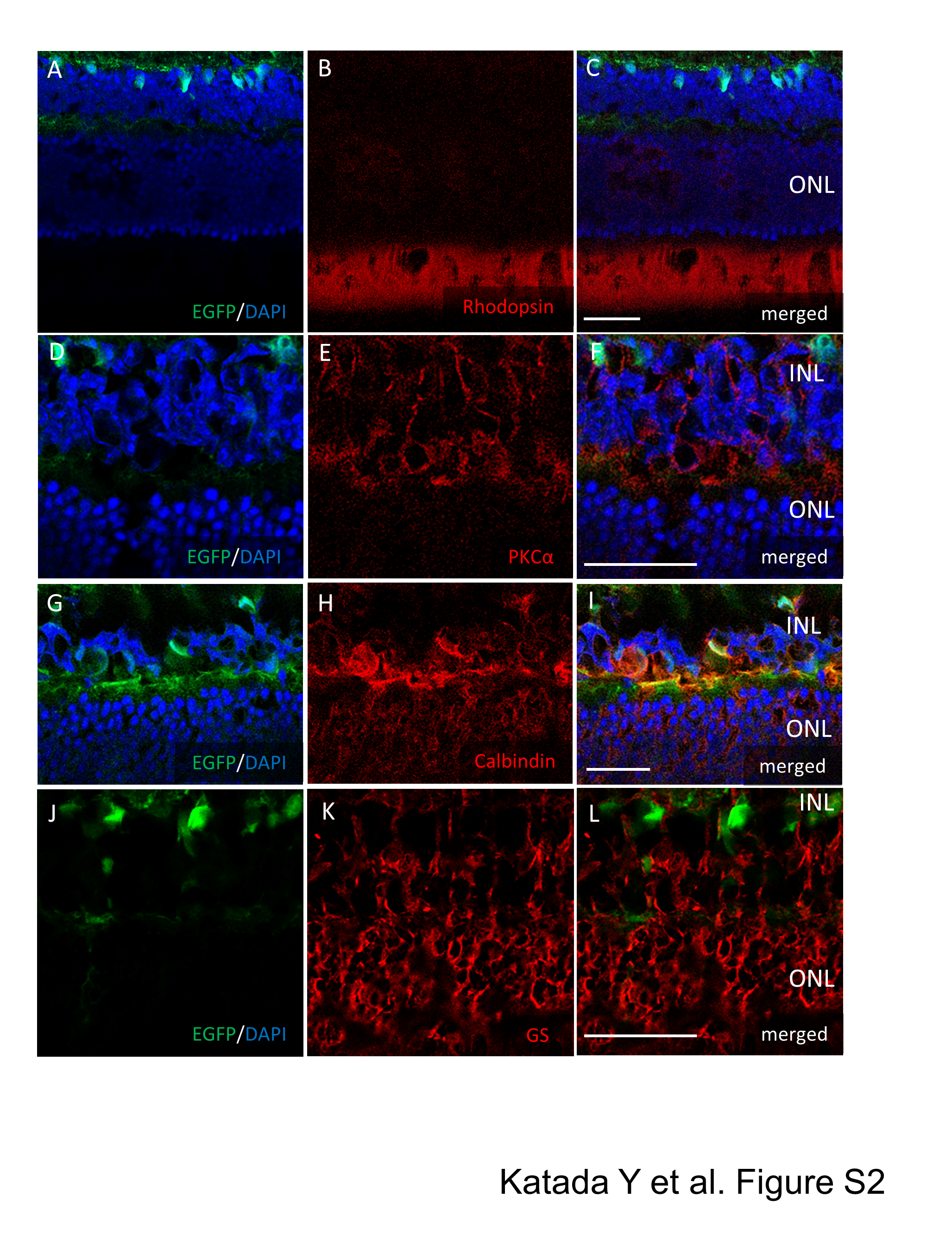

Supplement: Figure S2 — Immunohistochemistry on the transverse retinal cryosection with intravitreal injection of AAV-2-CAGGS-EGFP for (A–C) rod photoreceptors labelled with rhodopsin, (D–F) bipolar cells labelled with PKCα, (G–I) horizontal cells labelled with calbindin, and (J–L) Mueller cells labelled with GS. INL, inner nuclear layer; ONL, outer nuclear layer. Scale bars, 50 µm in A–L. [file peerj-07-6317-s002.png]
